# Intraregional propagation of Covid-19 cases in Pará, Brazil: assessment of isolation regime to lockdown

**DOI:** 10.1017/S095026882100039X

**Published:** 2021-02-16

**Authors:** Félix Lélis da Silva, Javier Dias Pita, Maryjane Dias A. Gomes, Andréa P. Lélis da Silva, Gabriel Lélis P. da Silva

**Affiliations:** 1 Federal Institute of Science and Technology of Pará – IFPA Campus Castanhal, Castanhal, Brazil; 2 Research Group for Biosystems Management, Modeling and Experimentation – GEMAbio, IFPA, Castanhal, Brazil; 3 Metropolitan Regional Hospital – Specialist in Intensive Care Unit, Pará, Brazil; 4 Central University of Paraguay, Medicine undergraduate student, Cidad del Leste, Paraguay

**Keywords:** Advance, contagion, control, coronavirus, syndrome

## Abstract

Due to the high incidence of COVID-19 case numbers internationally, the World Health Organization (WHO) declared a Public Health Emergency of global relevance, advising countries to follow protocols to combat pandemic advance through actions that can reduce spread and consequently avoid a collapse in the local health system. This study aimed to evaluate the dynamics of the evolution of new community cases, and mortality records of COVID-19 in the State of Pará, which has a subtropical climate with temperatures between 20 and 35 °C, after the implementation of social distancing by quarantine and adoption of lockdown. The follow-up was carried out by the daily data from the technical bulletins provided by the State of Pará Public Health Secretary (SESPA). On 18 March 2020, Pará notified the first case of COVID-19. After 7 weeks, the number of confirmed cases reached 4756 with 375 deaths. The results show it took 49 days for 81% of the 144 states municipalities, distributed over an area of approximately 1 248 000 km^2^ to register COVID-19 cases. Temperature variations between 24.5 and 33.1 °C did not promote the decline in the new infections curve. The association between social isolation, quarantine and lockdown as an action to contain the infection was effective in reducing the region's new cases registration of COVID-19 in the short-term. However, short periods of lockdown may have promoted the virus spread among peripheral municipalities of the capital, as well as to inland regions.

## Introduction

The COVID-19 pandemic stands out as the main global health crisis [1]. It started in Wuhan, China in December 2019 [2–4]. It is a respiratory infection caused by the coronavirus family (2019-nCoV) [5]. Its etiological agent is Sars-CoV-2 [6, 7], which causes severe acute respiratory syndrome (SARS) [8]. The viral infection presents severe clinical symptoms such as fever, dry cough, dyspnoea and pneumonia [1, 9] and can cause the death of the infected.

The transmission factors of a viral disease can be as variable as possible. The spread of a virus is associated with environmental factors (temperature, wind speed and relative humidity), population density, and the organisation and efficiency of the health sector, as well as general factors such as the biological characteristics of the virus, incubation time, effects on infected and susceptible people [9, 10]. The climate and other environmental factors are determinants in the infection, distribution and transmission of viruses, presenting different behaviour according to the region [11].

The main transmission mode of many viruses occurs through contact with infected people; however, some resistant viruses are released into the environment and persist on surfaces, water and air [12].

In the environment, the spread of the SARS-CoV-2 virus showed positive or negative correlations with several environmental factors [1, 5, 13]. However, the combinations among the levels of these environmental factors can promote or harm the dynamics of contamination, since the virus behaviour and the reaction with the environments is highly variable, depending on the climatic variation and the interaction of climatic factors of the environment of the involved region.

Epidemiological data associated with COVID-19 infection have shown different dynamics in several countries [14]. Several studies have shown the reverse effect of rising temperatures and the confirmed number of new cases [1, 15]. In Brazil, a country with a tropical climate, with an average annual temperature ranging from 16 to 27.4 °C, modelled results show a negative effect on the linear relationship of temperature with the new confirmed cases number [5].

Thus, it is of fundamental importance to study the infection of COVID-19 in the different climatic variations of the planet to know the dynamics of the evolution of cases in environments with different climatological interactions. For instance, in Brazil, a country with a tropical climate, the interactions of climatological factors in its different regions have a diversity of behaviour. Despite being considered a country of tropical climate, it has climatic peculiarities in its regions such as hot and extremely humid climate, dry and cold climate, dry and hot climate, among other climatic variations.

In addition to the climatic variations existing in the different regions of Brazil, other factors have contributed to pandemic advance in Brazil, as the country, in addition to having a deficiency in the number of doctors per inhabitant, the number of ICU beds and ventilators available for urgent and emergency cases, presents several risk groups, such as elderly over 60 years old, people with prognostic comorbidity, indigenous people and population's great genetic variation [14].

Due to the peculiarities of each place, the Government actions to mitigate the COVID-19 epidemic curve have been adopted in several countries. In Spain, measures of social distancing led to a cases curve flattening; after the first few days, the cumulative change rate in new cases decreased by an average of 3.059 percentage points daily [6].

In the northern region of Brazil, in Pará state, the government adopted social isolation; however, high mortality rates were reported, which raised the curve of new cases and directly affected hospital care due to overcrowding by infected people. On 6 May 2020, a more extreme control policy was decreed by the State of Pará government aimed at containing the pandemic advance, setting the lockdown model in the metropolitan region and neighbouring municipalities, trying to reduce the traffic of people and, consequently, increase the control of the pandemic advance.

People's traffic control measures are fundamental in controlling the pandemic progress. The cases reduction of COVID-19 in Wuhan observed in February 2020 coincided with travelling control measures adopted in the region [16].

Therefore, it has been suggested that the assessment of environmental factors and the social distancing tend to control the new cases and death numbers due to COVID-19, as well as the deficiency in the health system associated with the reduced number of intensive-care physicians, low numbers of mechanical ventilators, the minimum number of available beds and the specific medication absence tend to compromise the diagnosed population care. It is questioned: What is the dynamics of the evolution of new community cases and mortality records by COVID-19 in subtropical regions with temperatures between 20 and 35 °C, with social distance by quarantine and the adoption of lockdown?

## Materials and methods

### Study area

**Fig. 1.**
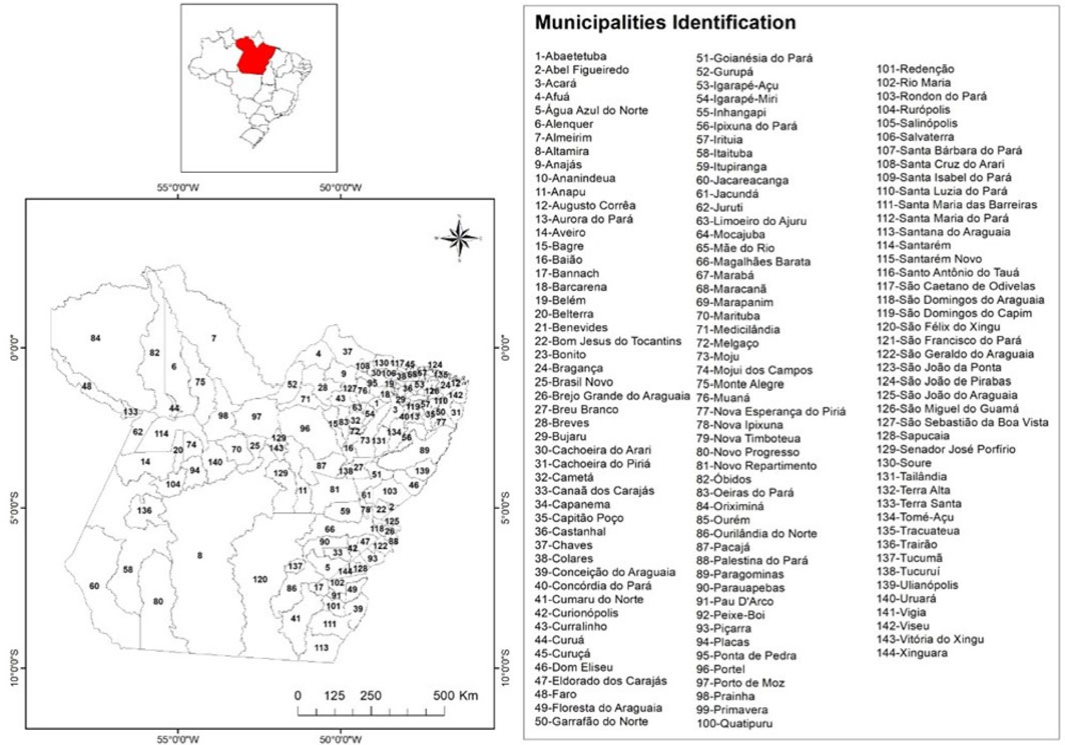
State of Pará, Northern Brazil and its 144 municipalities.

The study was carried out in 114 affected municipalities among the 144 existing in the State of Pará, North region, Brazil (Fig. 1). The state has an approximately territorial extension of 247 689 515 km^2^ and an estimated population of 8 602 285 people, with 8 191 559 residents in urban areas and 2 389 492 in rural areas, with a population density of 6.07 inhabitants/km^2^ and an average Human Development Index of (HDI = 0.698) [17].

### Data and sources

The cumulative daily number of deaths by age group and sex of COVID-19 cases in 114 municipalities in the State of Pará, with a record of infection, were obtained through the daily monitoring of technical bulletins provided by the State of Pará Public Health Secretary [18]. General data for Brazil and its northern region were obtained from the Secretary of Health Surveillance of the Ministry of Health [19].

### Study measures

The disease incidence in the studied region was understood through the cohort study. The dynamics of the frequency of new cases of COVID-19 in the populations of the inland regions made possible through community contamination during the epidemiological weeks was analysed through the ecological analytical epidemiological study.

### Data analysis procedure

Since understanding the dynamics of infection spread can favour more effective public control policies and allow the control of transmission in new regions, an exploratory data analysis was carried out, with numerical variables described using means, standard deviations, coefficient of variation, distributions and Pareto.

Weekly maps were built with the new cases accumulated, in the period of 49 days, to assess the spread of the COVID-19 pandemic in the municipalities of the State of Pará. To classify the evolution of the number of new cases, the Jenks algorithm based on the Absolute Deviations over the Median of Classes was used.

## Results and discussion

In Brazil, on 5 May 2020, 114 715 cases of COVID-19 and 7921 deaths were registered, with a lethality rate of 6.8%; the Southeast and Northeast regions are the most affected counting around 75% of the registered cases. The most affected regions in the country are the Southeast (64 756; 44.6%), followed by the Northeast (45 724; 31.5%) and North (23 207; 16.0%) [19]. In the North, the state of Amazonas had the largest number of confirmed infected, 10 727 cases and disease lethality around 874 registered deaths. The country on 6 May 2020 registered 114 715 cases and 7921 deaths.

In Northern Brazil, the federative units most affected on 5 May 2020, in terms of the spatial distribution of registered cases of COVID-19 with an incidence and mortality rate per 1 000 000 inhabitants, were Amazonas (2327.5 and 251.7) and Pará (627.4 and 49.5). The capital of Pará has the highest incidence (1816.4/1 000 000 inhabitants) and mortality (240/1 000 000 inhabitants) with a mortality rate of 9.9%.

Predictive studies in Brazil report smaller records of new cases of COVID-19 at temperatures around 25.8 °C, reducing the behaviour of curve growth [5]. SARS-CoV-2 may be vulnerable to fluctuations in environmental conditions similar to other coronaviruses [20]. It is important to note that climatological factors variability can interfere with the curve behaviour even at higher temperatures.

**Fig. 2.**
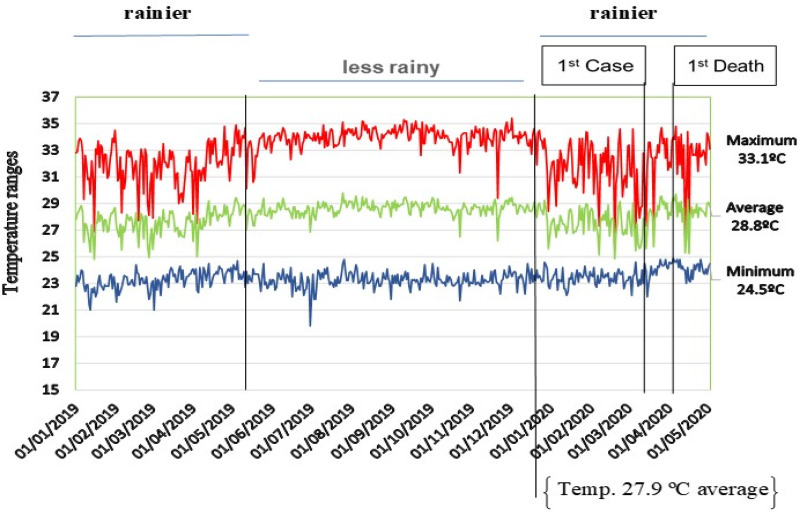
Temperature behaviour (°C), rainfall behaviour, first confirmed case and first death registration by COVID-19 in the State of Pará.

The northern region of Brazil has an equatorial climate ranging from humid to semi-arid, with temperatures ranging from 20 to 35 °C. In the State of Pará, the temperature presents spatial and seasonal homogeneity, with an average variation of 25–35 °C. In the region, there are two distinct periods of temperature ranges, classified as rainier between December and May and less rainy between June and November, according to rainfall variation that occurs in the Amazon. The metropolitan region of Belém city in the period between 1 January 2020 and 5 May 2020 presented temperature variation between 24 and 31 °C and relative humidity between 70% and 80%. In this period, the number of new cases and mortality due to COVID-19 has risen since the first case notification on 18 March 2020, with the first death recorded on 1 April 2020 (Fig. 2).

The North region was in third place in cases number with approximately 16% of the confirmed cases on 5 May 2020. In the State of Pará, the outbreak of COVID-19 registered a record of 4756 cases with an incidence rate of 788 cases in 49 days after the first infection confirmation. The first confirmed case of the disease was notified on 18 March 2020, in Belém city, capital of the state. These records were registered when the average temperature of the region, for the period considered to be the rainiest in the region, was 27.9 °C and the air relative humidity range was from 70% to 80%. The first notification of death occurred on 1 April 2020, and since then the curve of new cases and deaths incidence has been frequent and registered with high rates in the region.

Studies have suggested that temperature is a leading factor of COVID-19 [21], and acts as an indicator of SARS COV transmission, showing that the incidence is different in temperature variations in the region [22]. *A priori* it is suggested that there is no inverse relationship between ambient temperature and new cases of COVID-19 in Pará in the period considered as the rainiest in the Amazon region.

Therefore, there is no evidence to suppose that in regions with warm climates, low relative humidity and high rainfall variation, the cases number is lower compared to regions with moderate and/or cold climates. Similar results were obtained in the transmission rate evaluation of the new coronavirus in different provinces of Iran [23].

However, regions with a colder climate and high population density may have contributed to the spread of the disease in Europe [24]. Results obtained for Brazilian cities indicated negative behaviour for linear correlation between temperatures and numbers of confirmed cases [5]. Also, the climate is an important factor in determining the incidence rate of COVID-19, and the temperature and its variations are correlated with the infection records [25].

**Fig. 3.**
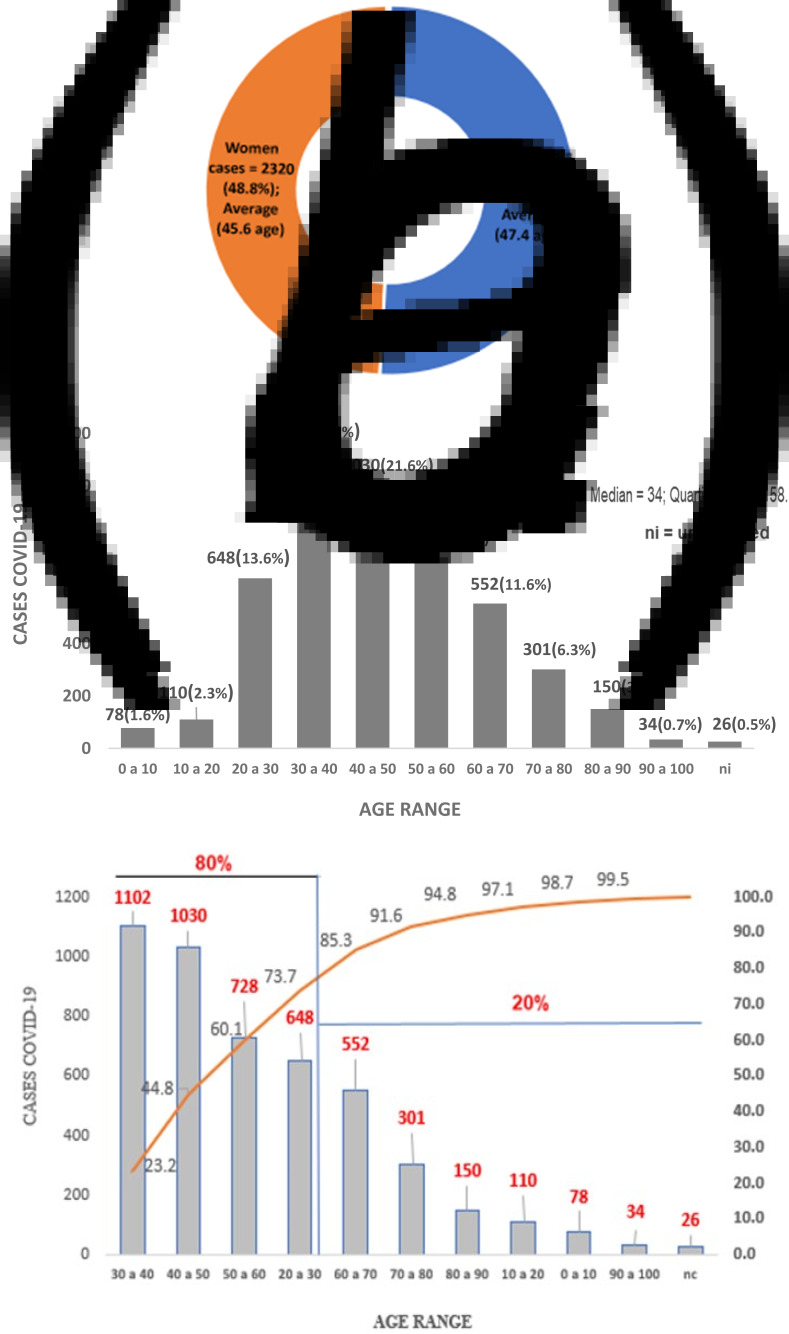
Exploratory statistical analysis of the confirmed cases accumulated of COVID-19 by sex (a), age group (b) and Pareto distribution of ages (c) of those infected, Pará-Brazil.

Figure 3 summarises the overall cases number compared to diagnosed population sex, the cumulative daily descriptive statistics of newly notified cases of COVID-19 and the age group distribution behaviour of the diagnosed population with the infection since the first disease case notified in the State of Pará.

The analysis was carried out after 7 weeks of the first COVID-19 case recorded in Pará, being 4756 cases reported between 18 March and 5 May 2020. In total, 2420 (50.9%) diagnosed cases were men and had an average age of 45.6 years. The women were 2320 (48.8%) infected cases with an average age of 47.4 years. It is reported that 15 registered cases did not present notification regarding gender and were considered as unidentified (Fig. 3a). Similar results of higher COVID-19 cases prevalence in men were obtained [26, 27].

In Pará, 75% were under 58 years old. Only 25% of infected cases were elderly and 1.6% of those diagnosed infected were children from 0 to 10 years old. Only 26 (0.5%) registered cases had no age identified and were considered in terms of registration as ‘unidentified’ (Fig. 3b).

In terms of the sample of the infected population age range, negative asymmetric behaviour was identified for distribution, with age concentration around the median of 34 years old, with 80% of cases diagnosed aged between 20 and 60 years old (Fig. 3c). Studies suggested for the region of the study that the population concentration and demographic density (inhabitants/km^2^) of the municipalities in the region have shown a great correlation with the daily record of cases of COVID-19 infection; however, the distribution of cases according to age group has not shown a disparity between men and women [28].

In Iran, the cases concentration was between 30 and 70 years old for 79.1% of COVID-19 records and approximately 39% for the elderly [27]. In the study region, 80% of the infections reported were recorded in age groups of economically active people (Fig. 3c), a factor that favoured the impact on the income generation of the families involved.

Covid-19 caused a significant global social and economic crisis. Social distancing and the blocking of people traffic caused serious demographic changes and unemployment [29]. On the global scenario, governments have introduced social blocking measures to ensure the security of their citizens [29].

The management of a country's pandemic involves multi and interdisciplinary treatment because the health crisis has systemic repercussions, and joint and integrated actions minimise the impact of the health crisis on the economy, politics and social life. In several countries, and in Brazil specifically, the epidemic crisis is revealing the most aggressive sides for society, and Pará State is no exception.

Concerned with the new cases index, dissemination to inland regions and increased mortality rates, the government of Pará established a decree for lockdown in the capital and additional nine municipalities to increase the social isolation index, reducing the disease spread and reducing the new cases registration number of COVID-19.

The restrictive measure was initially focused on forcing social isolation in the municipalities of Belém, Ananindeua, Marituba, Benevides, Santa Bárbara do Pará, Santa Izabel do Pará, Castanhal, which form the Belém Metropolitan Region (BMR) and inland towns, such as Santo Antonio do Tauá, Vigia de Nazaré and Breves. The lockdown arises when the State of Pará registered a COVID-19 rate in the order of 51/100 000 inhabitants, higher than the national rate. The municipalities affected by the restriction presented the rates of 75/100 000 inhabitants, higher than that registered by the state.

Despite the restrictions having focused on only 10 municipal regions, it is worth mentioning that when the lockdown was set, more than 70% of the municipalities forming the State of Pará already had notifications of COVID-19 cases.

The disease regional expansion and the high registration rates in the BMR have compromised the structure of the state's public and private healthcare systems due to the high demand for basic care services and the high need for more complex services involving hospitalisations and intubation of patients with aggravated cases.

**Fig. 4.**
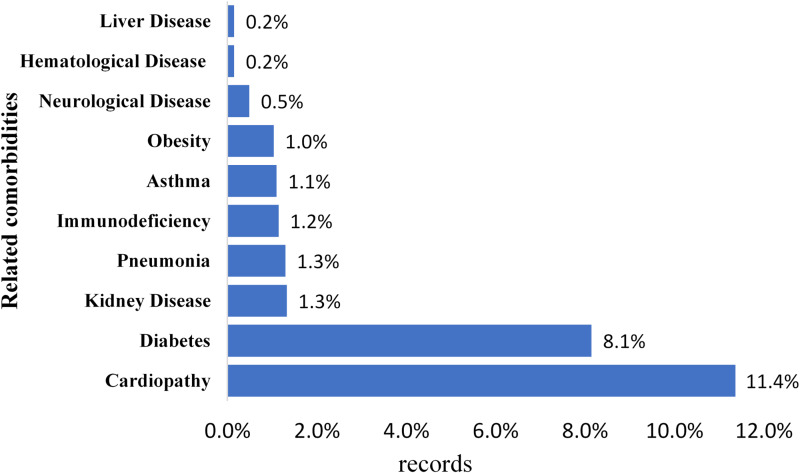
Common comorbidities in diagnosed cases of COVID-19 in the State of Pará, from 18 March to 5 May 2020.

In the Pará state, severe cases of COVID-19 and mortality records are related to several comorbidities, the most commons are associated with heart disease, diabetes, kidney disease, pneumonia, immunodeficiency, asthma, obesity, neurological disease, haematological disease and illness hepatic; the most frequent ones being associated with heart disease and diabetes (Fig. 4). Patients infected with COVID-19 tend to have associated comorbidities with one or more chronic diseases [26].

In Iran, patients diagnosed with COVID-19 had a strong association with chronic diseases such as diabetes, chronic respiratory diseases, hypertension, cardiovascular diseases, kidney diseases and cancer; these comorbidities were also observed in cases of deaths [27]. Similar results were verified in infected people in Wuhan, China, corroborating with the results that occurred in this study region [1].

These factors associated with the precarious service capacity of hospitals and emergency care units (ECU), the insufficient health professional staff and lack of rapid tests to identify infected patients contributed to COVID-19 advance in the region. Associated with these problems, there is still an insufficient hospital bed number available [18]. There are only 249 ICU beds available for adults care in the pandemic for a population of 7 581 051 inhabitants, which represents a ratio of 1/30 445.98 bed/inhabitants, such fact leads the system to collapse in a short period during a pandemic, contributing to the disease and the number of deaths progress. Collapsing is already experienced in paediatric clinic beds (Table 1).

**Table 1.** Availability and occupation of exclusive hospital beds for COVID-19 in the public healthcare system in the State of Pará

Hospital bed type	Total	Available	% Occupation
Paediatric	1109	497	55.2
Paediatric clinical	8	8	0.0
Adult ICU	249	37	85.2
Paediatric ICU	7	4	42.9
Intermediate unit	26	25	3.9
Neonatal ICU	4	1	75.0

*Source*: Secretary of Public Health of Pará. https://portalarquivos.saude.gov.br/images/pdf/2020/May/09/2020-05-06-BEE15-Boletim-do-COE.pdf

Due to the risk of COVID-19-infected people promoting the disease advance through community transmission to more remote regions of the state, it is essential to understand the dynamics of new cases of the disease to enable decision-making regarding control, prevention, treatment of infected, identify groups of risks and enable decision-making on resources allocation and allow better planning on the health system in difficult times.

**Fig. A1.**
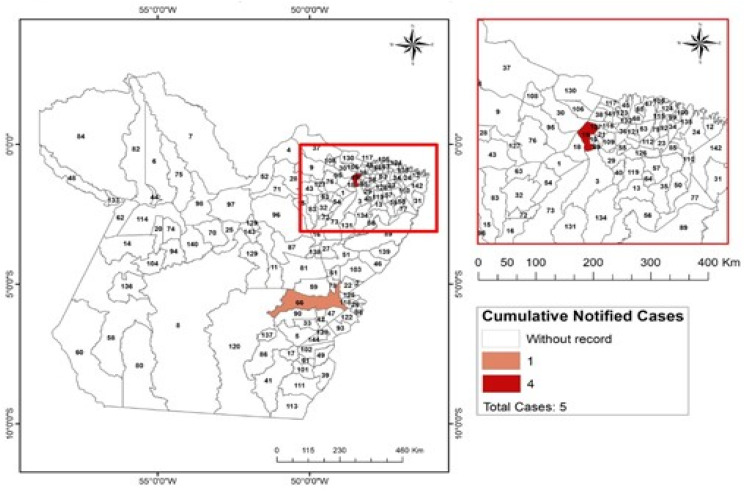
Propagation of new cases of COVID-19 accumulated in the municipalities of the State of Pará in the first week after registration of the first case in the region.

**Fig. A2.**
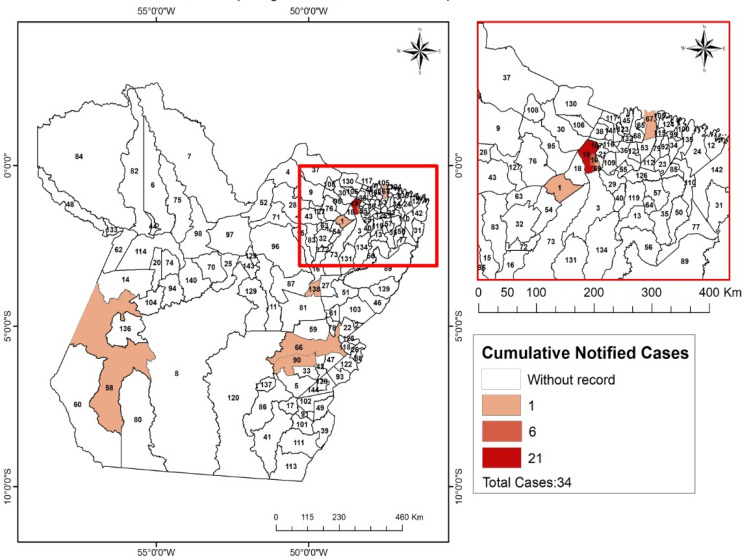
Propagation of new cases of COVID-19 accumulated in the municipalities of the State of Pará in the second week after registration of the first case in the region.

**Fig. A3.**
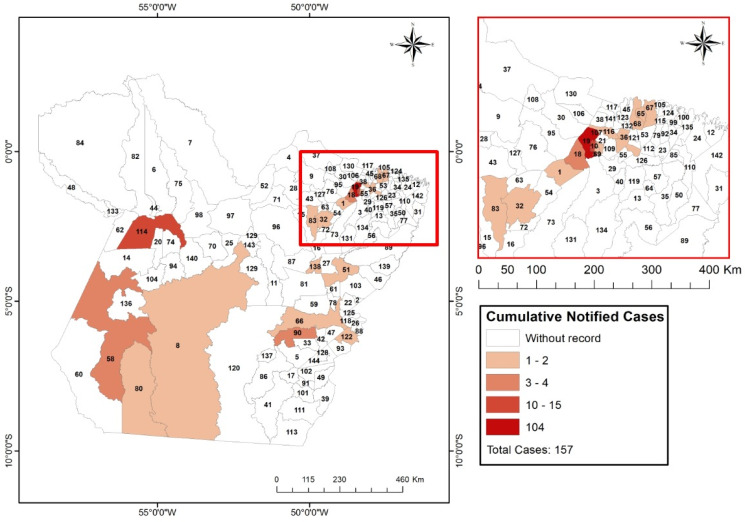
Propagation of new cases of COVID-19 accumulated in the municipalities of the State of Pará in the third week after registration of the first case in the region.

**Fig. A4.**
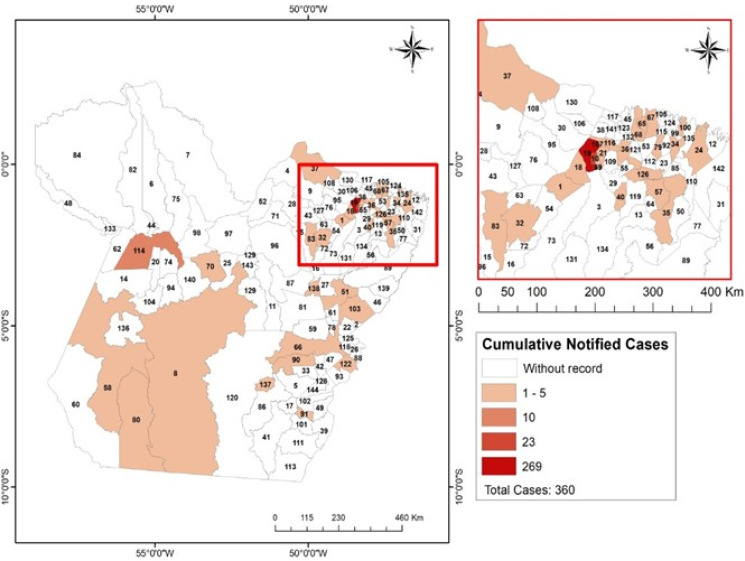
Propagation of new cases of COVID-19 accumulated in the municipalities of the State of Pará in the fourth week after registration of the first case in the region.

**Fig. A5.**
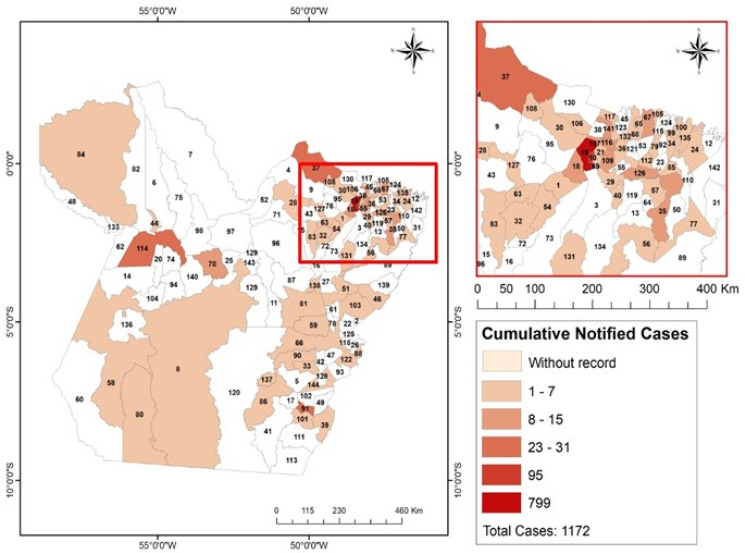
Propagation of new cases of COVID-19 accumulated in the municipalities of the state of Pará in the fifth week after registration of the first case in the region.

**Fig. A6.**
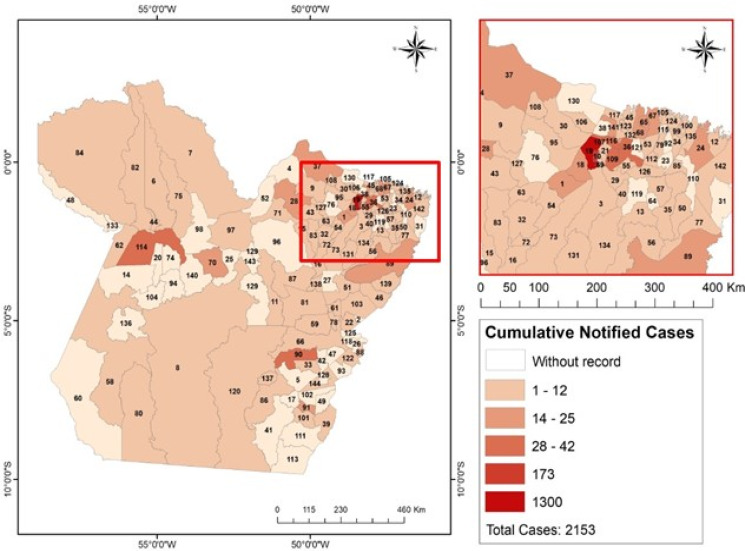
Propagation of new cases of COVID-19 accumulated in the municipalities of the state of Pará in the sixth week after registration of the first case in the region.

**Fig. A7.**
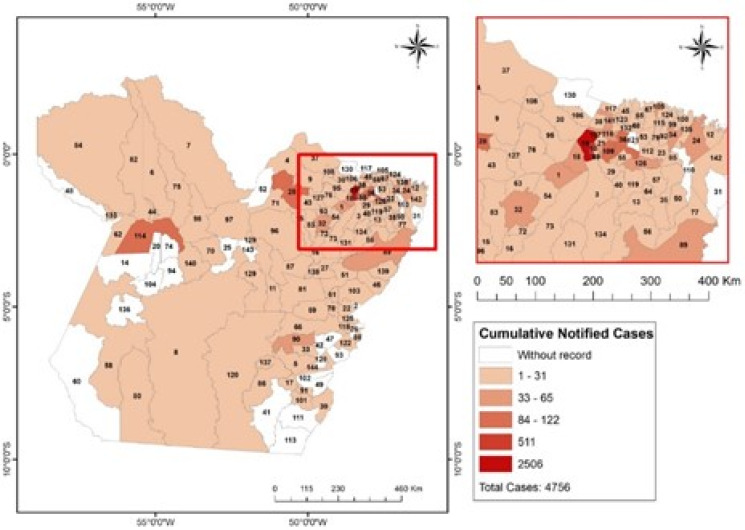
Propagation of new cases of COVID-19 accumulated in the municipalities of the state of Pará in the seventh week after registration of the first case in the region.

Results show that a week after the first notified case, only 0.82% of the municipalities had infected people, in the second week of contagion they already had 5.56%, in the third 15.3%, in the fourth 24.3%, in the fifth 46.5%, in the sixth 69.4% and the seventh week after the first notified case, there were already 81.8% of the municipalities in the State of Pará with notified COVID-19 cases (Fig. A1-A7). The highest concentration of registered cases was in the Metropolitan Region of Belém, constituted by the capital Belém, Ananindeua, Marituba, Benevides, Santa Bárbara do Pará, Santa Izabel do Pará, Castanhal.

**Fig. 5.**
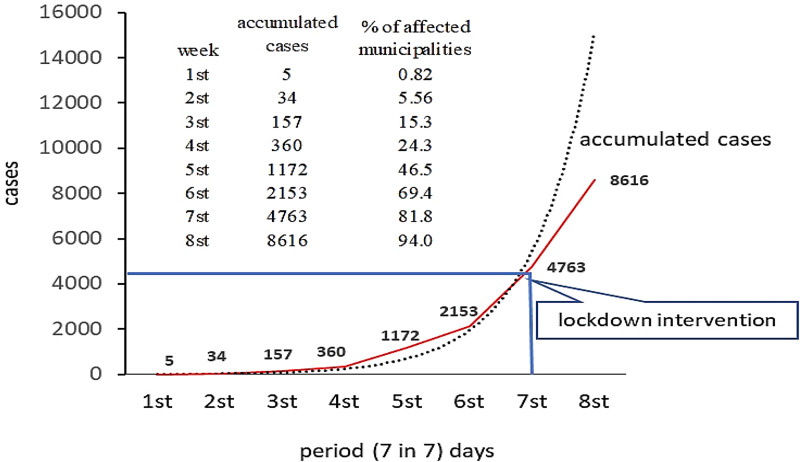
Propagation of notified COVID-19 cases in the municipalities of the State of Pará, from 18 March to 5 May 2020, and the first post-lockdown period.

The measure of containment by social isolation in the region did not have an expected effect on the curve of new cases for the period under study, mainly due to the low rate of population adherence. However, the lockdown instituted on 5 May 2020 gave positive percentage results in the short-term on new cases registration in the following week of decreed intervention, with a 10.07% reduction compared to the accumulated record in the previous week (Fig. 5). In the long-term, the reduction of the cases may be more significant, given the greater respect and adherence of the population to isolation policies.

Blockades when needed in epidemic situations are critical to strengthening health systems as they reduce pressure on the system and prevent overload. However, when evaluating different scenarios involving roadblocks, it was suggested that a COVID-19 mitigation policy based on blockade should be well defined and practised, considering some precautions such as population, economy, transportation and the health system of the region, as these factors affect the levels of contact between people [30]. The author adds that blockade in small towns can be highly damaging.

Social control through lockdown in Pará was limited, due to the municipalities of the metropolitan region presenting a high level of informality, rates above 58%, which means that isolation measures in a reality of high demand for daily money earning do not allow financial planning for groups of high socioeconomic vulnerability [28].

The epidemiological crisis caused by SARS-CoV-2 had serious consequences in several sectors of the economy and favoured unemployment with the closure of several activities; however, these measures as a way to contain the epidemic around the world were crucial to saving human lives [31].

As a consequence, the low adherence to isolation reinforces the current health crisis in a trend of continuity, considering that the prevalence is still low in the population of the State, far from a collective immunisation and without a possible vaccine. An effective way to experience the drop in the records of COVID-19 cases in the region is suggested a longer period of blockade combined with greater adherence and consciousness of the population, understanding that such measures are essential to control the pandemic. It is worth mentioning that a gradual and well-defined policy of unblocking, combined with population monitoring and the records of new cases for immediate containment, can prevent the spread of SARS-CoV-2 infection and deaths due to COVID-19.

Despite not having the expected effect on the cases curve, several studies have confirmed that the blockades, due to acting on human traffic, have considerably reduced environmental problems linked to air pollution [31–33], which has contributed to public health and the environment as the overload of atmospheric pollution has been reduced, making the environment healthier.

In this regard, the climatic conditions of the region and the concentration of air pollutants are correlated with the epidemics of viral bronchitis [34]. Studies suggest that there is a significant correlation between air quality and COVID-19 cases, since chronic exposure to environments with high loads of atmospheric pollutant may favour the spread of SARS-CoV-2 [35], corroborating with studies that suggest a direct relationship between the spread and the contagion capacity of some viruses with atmospheric levels and the mobility of air pollutants [36].

Also, chronic exposures to contaminated atmospheric environments can represent a risk factor for COVID-19 and the high incidence of fatal events [14, 24, 37]. It was recommended that in cities located in inland regions that present air pollution and high levels of particulate compound emissions in the atmosphere, low wind intensity and lower temperatures, the number of notifications of infected people was higher compared to other regions [38].

The adoption of strict blocking measures allowed to reduce the impact of the Covid-19 pandemic on the National Health System in Italy, along with the imposed lockdown allowed to evaluate the effect of human activities on the emission of atmospheric pollutants [39].

Efforts to prevent infected people from reaching more remote regions are important to stop disease transmission and prevent new cases [9]. In this context, control policies based on screening through the wide use of rapid tests, in order to early diagnose infected people at the entrance or exit of countries and regions, considering that the diagnosis and rapid identification of infected people may interrupt the contamination among people, prevent new infections and anticipate specific health treatment for these people [31].

To understand the dynamics of COVID-19 cases advance to remote regions from the capital, seven maps were adjusted and show the weekly evolution of the accumulated cases registered in the municipalities of the state (Appendix: Figs A1 to A7).

The municipalities that registered cases of infection in the first week were the capital Belém (code 19) with two cases and Marabá (code 67) with one notified case; in both cases, the contamination was motivated by the traffic of infected people from large urban centres, where community contamination was in the expansion (Appendix: Fig. A1).

The inland regions that did not present COVID-19 case records in the 7 weeks after the first case were: Abel Figueiredo (code 2), Aveiro (code 14), Belterra (code 20), Brasil Novo (code 25), Brejo Grande do Araguaia (code 26), Cachoeira do Arari (code 30), Cumaru do Norte (code 41), Curionópolis (code 42), Eldourado dos Carajas (code 47), Faro (code 48), Araguaia Forest (code 49), Gurupá (code 52), Jacareacanga (code 60), Mojuí dos Campos (code 74), Piçarra (code 93), Plates (code 94), Rio Maria (code 102), Rurópolis (code 104), Santa Luzia do Pará (code 112), Santa Maria das Barreiras (code 111), Santana do Araguaia (code 113) Francisco do Pará (code 121), Sapucaia (code 128), Soure (code 130), Trairão (code 136) and Vitória do Xingu (code 120) (Appendix: Fig. A7).

These results suggest that regions further away from the capital and with low people density took longer to register the occurrence of infections, proving that in the region, the dissemination process took place through the person-to-person contact. Thus, the absence of a longer period of blockade and/or policies of gradual unblocking favoured the spread of the intraregional virus in the State of Pará.

## Conclusions and limitations

The social isolation and quarantine combined with the adoption of a strict measure of population circulation, the ‘Lockdown’, and the mandatory use of masks in public environments were effective in reducing the new cases of COVID-19 registration in the short-term.

Temperature variations between 24.5 and 33.1 °C with an average of 28.8 °C did not show any reduction in the records of new cases. However, studies with multiple associations between environmental variables are essential in order to understand the multiple correlations (temperature, wind, relative humidity, rainfall, radiation) on the occurrence of new records in the region.

The results are extremely necessary for decision-making and public health planning to combat the spread and control of the disease in poor regions of the State of Pará.

However, due to the territorial dimension of the State and the difficulty of accessing certain regions, this study is limited by the substantial proportion of data not reported or late reported in some municipalities. The lack of rapid mass testing in populations and a broader lockdown period was the driving force for dissemination to inland regions, as these actions would allow greater control of those infected and a better understanding of the spread of diseases in the State and less bias in the calculation of incidence of the disease in the population.

## Data Availability

The data supporting the results of this study are openly available on government websites such as the Ministry of Health of Brazil at https://covid.saude.gov.br/and https://portalarquivos.saude.gov.br/images/pdf/2020/May/09/2020-05-06-BEE15-Boletim-do-COE.pdf. Likewise, at the state level at the Secretary of State for Health of Pará-Brazil (SESPA-PA) at http://www.saude.pa.gov.br/coronavirus/.
