# Intraregional propagation of Covid-19 cases in Pará, Brazil: assessment of isolation regime to lockdown– CORRIGENDUM

**DOI:** 10.1017/S0950268821000765

**Published:** 2021-04-22

**Authors:** Félix Lélis da Silva, Javier Dias Pita, Maryjane Diniz A. Gomes, Andréa P. Lélis da Silva, Gabriel Lélis P. da Silva

**Affiliations:** 1 Federal Institute of Science and Technology of Pará–IFPA Campus Castanhal, Castanhal, Brazil; 2 Research Groupfor Biosystems Management, Modeling and Experimentation–GEMAbio, IFPA, Castanhal, Brazil; 3 MetropolitanRegional Hospital–Specialist in Intensive Care Unit, Pará, Brazil; 4 Central University of Paraguay, Medicineundergraduate student, Cidad del Leste, Paraguay

This article was originally published with a mistake in the third author's name. The correct full name should be “Maryjane Diniz A. Gomes”. This has now been corrected in the online version of the article.

There were also several numerical errors in the paragraph titled “Study Area”. The corrected version of this paragraph is shown in full below.

The study was carried out in 114 affected municipalities among the 144 existing in the state of Pará, North region, Brazil (Figure 1). The state has a territorial extension of 1,247,698.515 km^2^ and an estimated population of 7,581,051 people, with 5,191,559 residents in urban areas and 2,389,492 in rural areas, with a population density of 6.07 inhabitants/km^2^ and an average Human Development Index of (HDI = 0.698) (IBGE, 2020).
